# A Series of Zr-Based Bulk Metallic Glasses with Room Temperature Plasticity

**DOI:** 10.3390/ma9060408

**Published:** 2016-05-25

**Authors:** Anhui Cai, Dawei Ding, Yong Liu, Hong Wu, Weike An, Guojun Zhou, Yun Luo, Yongyi Peng

**Affiliations:** 1 College of Mechanical Engineering, Hunan Institute of Science and Technology, Yueyang 414000, China; Anweike12@163.com (W.A.); gjzhoumike@163.com (G.Z.); luoyun810812@yahoo.com.cn (Y.L.); 2 State Key Laboratory of Powder Metallurgy, Central South University, Changsha 410083, China; yonliu@mail.csu.edu.cn (Y.L.); wuhong927@126.com (H.W.); 3 Institute of Physics, Chinese Academy of Sciences, Beijing 100190, China; dingdawei@iphy.ac.cn; 4 School of Physics and Electronics, Central South University, Changsha 410083, China; pengyongyi@126.com

**Keywords:** Zr-based bulk metallic glass, plasticity, mechanical property

## Abstract

A group of plastic Zr-Al-Ni-Cu bulk metallic glasses (BMGs) with low Zr content was developed and their thermal and mechanical properties were investigated. The results show that these Zr-based BMGs have a single crystallization event for all heating rates in the studied temperature region. The glass transition temperature *T*_g_ decreases with increasing Zr content for all heating rates. There are two melting procedures for the BMGs whose Zr content is less than 52 at %, while three melting procedures for the other Zr-based BMGs. The second melting procedure is split into two melting procedures for Zr_52.5_Al_12.2_Ni_12.6_Cu_22.7_ and Zr_53_Al_11.6_Ni_11.7_Cu_23.7_ BMGs, while the first melting procedure is split into two melting procedures for the other BMGs. The activation energy decreases with increasing sensitivity index β for the studied Zr-based BMGs. The plastic strain ε_p_ is in the region of 0.2%–19.1% for these Zr-based BMGs. Both yield strength σ_y_ and fracture strength σ_f_ are smallest for Zr_55_Al_8.9_Ni_7.3_Cu_28.8_ BMG whose ε_p_ is largest among all studied Zr-based BMGs and reaches up to 19.1%. In addition, the mechanism for the large difference of the plasticity among the studied Zr-based BMGs is also discussed.

## 1. Introduction

Zr-based bulk metallic glasses (BMGs) have been extensively investigated due to their high glass forming ability (GFA), large compositional region for glass formation, good thermal stability, and high strength [1,2,3,4,5,6,7,8,9,10]. In addition, some Zr-based BMGs have been found to be characterized by the combination of good mechanical and corrosive properties and good biocompatibility [9,10,11,12], which would ensure their potential applications as biomaterials and structural materials.

Room-temperature plasticity is one of important requirements for the engineering applications of the BMGs. Majority of Zr-based BMGs are room-temperature brittle although good room-temperature plasticity could be achieved through compositional design [3,4,5,6,7,8,9,10], minor addition [13], cold-rolling [14,15] and sand blasting [16]. For example, the room-temperature compression plasticity of Zr_70_Ni_16_Cu_6_Al_8_ and Zr_70_Al_8_Cu_13.5_Ni_8.5_ BMGs can reach up to 40% [6] and 70% [4], respectively. More interestingly, Liu *et al.* [3] developed three Zr-Al-Ni-Cu BMGs with super plasticity by carefully adjusting the composition. In addition, critical diameter (*d*_c_) for the formation of amorphous state is another crucial factor for engineering applications of the BMGs. However, increasing efforts have claimed that there is a discrepancy between the plasticity and the *d*_c_ for the BMG [17]. For example, the *d*_c_ of Zr_55_Al_10_Ni_5_Cu_30_ glass forming alloy can reach up to 30 cm [2], but its room-temperature plasticity is nearly zero [18,19]. Although the super plasticity can be achieved in Zr_61.88_Cu_18_Ni_10.12_Al_10_, Zr_64.13_Cu_15.75_Ni_10.12_Al_10_ and Zr_62_Cu_15.5_Ni_12.5_Al_10_ BMGs, their *d*_c_s are only 5 mm [3]. Thus, it is a significant and challenging work for how to design Zr-Al-Ni-Cu BMGs with the combination of large *d*_c_ and good room-temperature plasticity.

In the present work, a group of Zr-Al-Ni-Cu BMGs with room-temperature plasticity was carefully designed. It is found that the critical dimension and maximum plastic strain of the studied Zr-based BMGs can reach up to 8 mm and 19.1%, respectively. The present design concept would be a useful strategy for developing the BMGs with high GFA and plasticity.

## 2. Experimental

### 2.1. Compositional Design

A method, namely weak constraint condition proposed by Cai *et al.* [20], was used to design a group of Zr-Al-Ni-Cu BMGs whose *d*_c_ could reach up to 7.5 mm [20]. In addition, Cai *et al*. [20,21,22] found that e/a = ∑*C*_i_ × *E*_i_ (*C*_i_ and *E*_i_ are atomic fraction and conduction electron concentration of *i*-th element, respectively), *R*_a_ = ∑*C*_i_ × *R*_i_ (*R*_i_ is atomic radius of *i*-th element) and Δ*H*_m_ = ∑*C*_i_ × Δ*H*_mi_ (Δ*H*_mi_ is melting heat of *i*-th element) were strongly related with glass forming ability (GFA) of glass forming alloys. Recently, Cai *et al*. [23] developed a group of Zr-based BMGs with network structure by using the weak constraint condition method. Thus, the studied Zr-Al-Ni-Cu BMGs were developed by using the conditions of e/a = 1.38, *R*_a_ = 1.496 nm, ∑*C*_i_ = 1, and Δ*H*_m_ ≈ 19 kJ·mol^−1^. In addition, artificial neural network models were used to further optimize the compositions of these Zr-based BMGs [24]. The resultant compositions (in at %) of the studied Zr-Al-Ni-Cu glass forming alloys were listed in Table 1.

### 2.2. Materials and Methods

Pre-alloyed Zr-Al-Ni-Cu ingots with nominal compositions (see Table 1) were prepared by arc melting mixtures of Zr (99.99 wt %), Ni (99.99 wt %), Cu (99.99 wt %), and Al (99.99 wt %) in the Ti-gettered high purity argon atmosphere. Φ2 and Φ8 mm rods were prepared by suction casting into a water-cooled copper mold. The structures of the *as-cast* and DSC-tested samples were characterized by X-ray diffraction (XRD) using an X’ Pert Pro MPD diffractometer with Cu-*K*α (Holland Philips Company, Eindhoven, The Netherlands). The thermal analysis was conducted by DSC-404C differential scanning calorimeter (NETZSCH-Gerätebau GmbH, Bavarian State, Germany) at heating rates of 5–80 K∙min^−1^, respectively. Room-temperature uniaxial compression tests were performed on Φ2 × 4 mm BMGs with a gauge aspect ratio of 2:1 using an Instron 3369 testing machine (Instron Corporation, Norwood, GA, USA) at a strain rate of 1 × 10^−5^ s^−1^. Two polished end surfaces of the samples for the compression tests were parallel each other and vertical to the symmetry axis. Fracture morphologies of Φ2 mm samples were examined by SIRION 200 scanning electron microscopy (SEM, Holland FEI Company, Eindhoven, The Netherlands). It should be noted that at least three samples for all studied BMGs were examined in order to obtain reliable results.

## 3. Results

**Figure 1:**
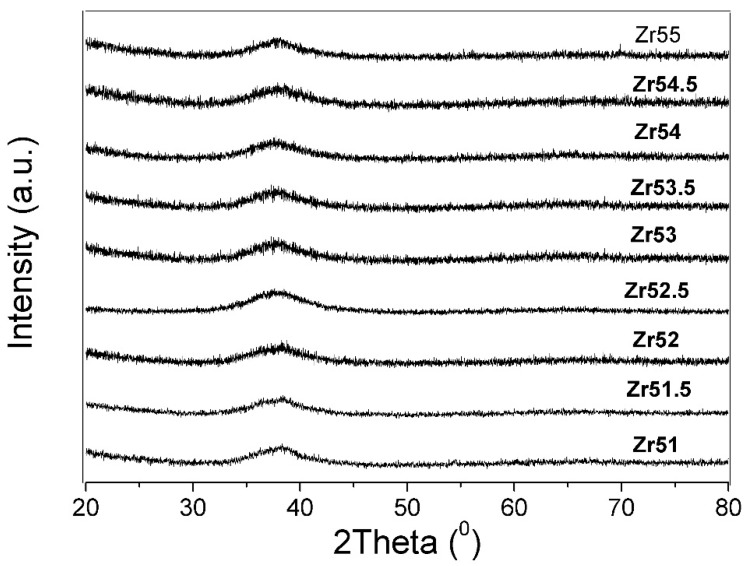
XRD patterns for Φ8 mm Zr-Al-Ni-Cu bulk metallic glasses.

Figure 1 presents XRD patterns for Φ8 mm Zr-based glass forming alloys, which indicates that these Φ8 mm Zr-based glass forming alloys are in glassy states because no apparent crystalline Bragg peaks are observed.

**Figure 2:**
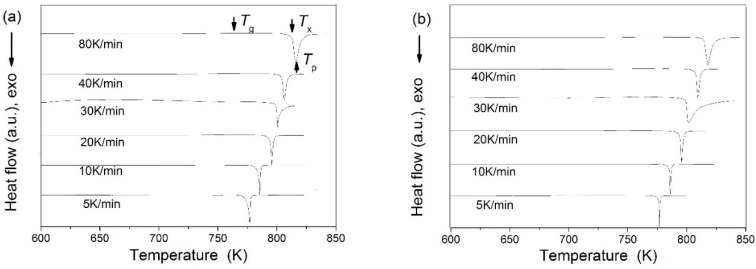

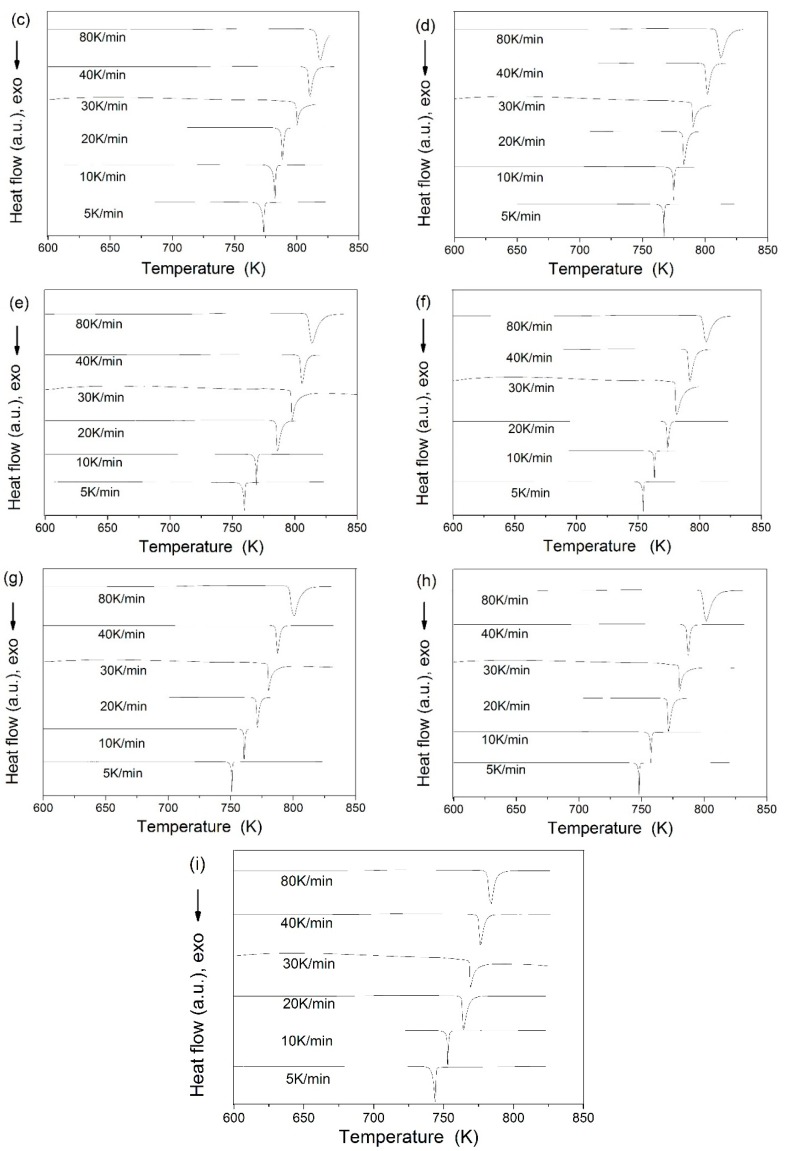
DSC curves for Φ2 mm Zr-Al-Ni-Cu bulk metallic glasses at heating rates of 5–80 K∙min^−1^: (**a**) Zr_51_Al_14.2_Ni_15.9_Cu_18.9_ BMG; (**b**) Zr_51.5_Al_13.6_Ni_14.9_Cu_20_ BMG; (**c**) Zr_52_Al_12.9_Ni_13.8_Cu_21.3_ BMG; (**d**) Zr_52.5_Al_12.2_Ni_12.6_Cu_22.7_ BMG; (**e**) Zr_53_Al_11.6_Ni_11.7_Cu_23.7_ BMG; (**f**) Zr_53.5_Al_10.9_Ni_10.6_Cu_25_ BMG; (**g**) Zr_54_Al_10.2_Ni_9.4_Cu_26.4_ BMG; (**h**) Zr_54.5_Al_9.6_Ni_8.4_Cu_27.5_ BMG and (**i**) Zr_55_Al_8.9_Ni_7.3_Cu_28.8_ BMG.

It is obvious from the DSC curves, as shown in Figure 2, that both glass transition and sharp crystallization events can be clearly observed at heating rates of 5–80 K∙min^−1^. It further confirms the glassy nature of these Zr-based BMGs. In addition, there is a single crystallization event in the studied temperature region for all heating rates, indicating one step crystallization procedure for all Zr-based BMGs.

**Figure 3:**
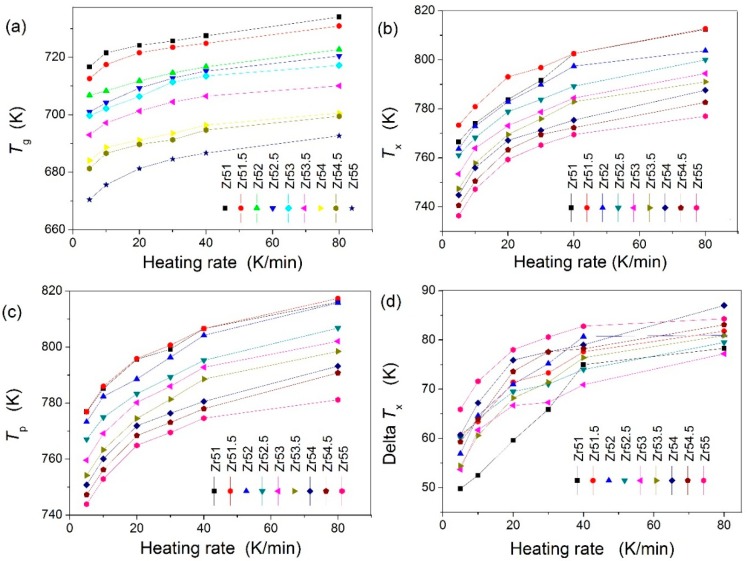
Dependences of the characteristic temperatures on the heating rates: (**a**) *T*_g_; (**b**) *T*_x_; (**c**) *T*_p_; and (**d**) Δ*T*_x_.

The characteristic temperatures including glass transition temperature (*T*_g_), crystallization temperature (*T*_x_), crystallization peak temperature (*T*_p_) and supercooled liquid region (Δ*T*_x_ = *T*_x_ − *T*_g_) were carefully determined according to the DSC results (see Figure 2). The dependences of the characteristic temperatures on the heating rate are shown in Figure 3. These characteristic temperatures all increase with increasing heating rate for the studied Zr-based BMGs. The *T*_g_ decreases with increasing Zr content for all heating rates, as shown in Figure 3a. Both *T*_x_ and *T*_p_ firstly increase with increasing Zr content and then decrease when the Zr content exceeds 51.5 at % for all heating rates, as shown in Figure 3b,c. However, there are complex relationships between the Δ*T*_x_ and the Zr content for the studied heating rates, as shown in Figure 3d. The Δ*T*_x_ for Zr_51_Al_14.2_Ni_15.9_Cu_18.9_ BMG is the smallest among the studied Zr-based BMGs when the heating rate is less than 30 K∙min^−1^. When the heating rate exceeds 30 K∙min^−1^, the Δ*T*_x_ for Zr_53_Al_11.6_Ni_11.7_Cu_23.7_ BMG is the smallest among the studied Zr-based BMGs. The Δ*T*_x_ for Zr_55_Al_8.9_Ni_7.3_Cu_28.8_ BMG is the largest among the studied Zr-based BMGs when the heating rate is less than 40 K∙min^−1^. Zr_54_Al_10.2_Ni_9.4_Cu_26.4_ BMG has the largest Δ*T*_x_ among the studied Zr-based BMGs at a heating rate of 80 K∙min^−1^.

**Figure 4:**
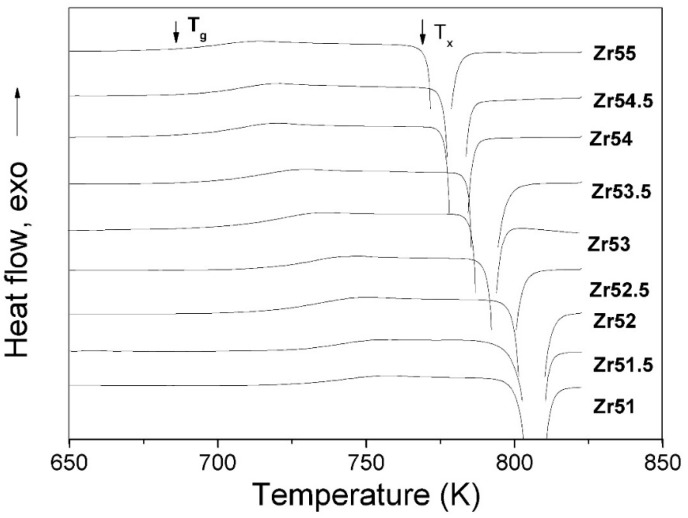
DSC curves of all studied Zr-based BMGs at a heating rate of 30 K∙min^−1^.

Figure 4 presents DSC curves of all studied Zr-based BMGs at a heating rate of 30 K∙min^−1^ for clear comparison and the corresponding thermal parameters are listed in Table 1. As shown in Figure 4, an endothermal event for the glass transition procedure can be clearly seen for all studied Zr-based BMGs. The *T*_g_ decreases with increasing Zr content. The *T*_x_ slightly increases and then decreases with increasing Zr content when the Zr content exceeds 51.5 at %. Table 1 lists the average crystallization enthalpy (Δ*H*_x_) for the studied Zr-based BMGs at heating rates of 5–80 K∙min^−1^. The Δ*H*_x_ is smallest for Zr_55_Al_8.9_Ni_7.3_Cu_28.8_ BMG and largest for Zr_52.5_Al_12.2_Ni_12.6_Cu_22.7_ BMG among the studied Zr-based BMGs.

**Figure 5:**
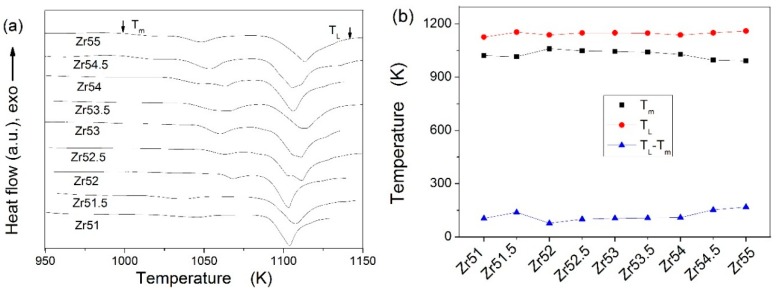
Melting curves at a heating rate of 30 K∙min^−1^ for the studied Zr-based BMGs (**a**); and dependences of the *T*_m_, *T*_L_, and *T*_L_-*T*_m_ on the Zr content (**b**).

In addition, the melting curves at a heating rate of 30 K∙min^−1^ are shown in Figure 5a. There are two melting procedures when the Zr content is less than 52 at %, while three melting procedures for the other Zr-based BMGs. It is well known that the eutectic alloy characterizes in a single melting procedure [25]. Figure 5a indicates that the studied Zr-based alloys are all off-eutectic compositions. The distance of two melting procedures is larger for Zr_51_Al_14.2_Ni_15.9_Cu_18.9_ and Zr_51.5_Al_13.6_Ni_14.9_Cu_20_ BMGs than for Zr_52_Al_12.9_Ni_13.8_Cu_21.3_ BMG. The second melting procedure is split into two melting procedures for Zr_52.5_Al_12.2_Ni_12.6_Cu_22.7_ and Zr_53_Al_11.6_Ni_11.7_Cu_23.7_ BMGs, while the first melting procedure is split into two melting procedures for the other BMGs. The values of the solidus temperature (*T*_m_) and liquidus temperature (*T*_L_) are listed in Table 1. The dependences of the *T*_m_, *T*_L_ and *T*_L_-*T*_m_ on the Zr content are also presented in Figure 5b. The *T*_m_ decreases with increasing Zr content when the Zr content is less than or exceeds 52 at %, while inversely for the *T*_L_-*T*_m_. However, there is a complex dependence of the *T*_L_ on the Zr content. The *T*_L_ is largest for Zr_55_Al_8.9_Ni_7.3_Cu_28.8_ BMG and smallest for Zr_51_Al_14.2_Ni_15.9_Cu_18.9_ BMG among the studied Zr-based BMGs.

**Figure 6:**
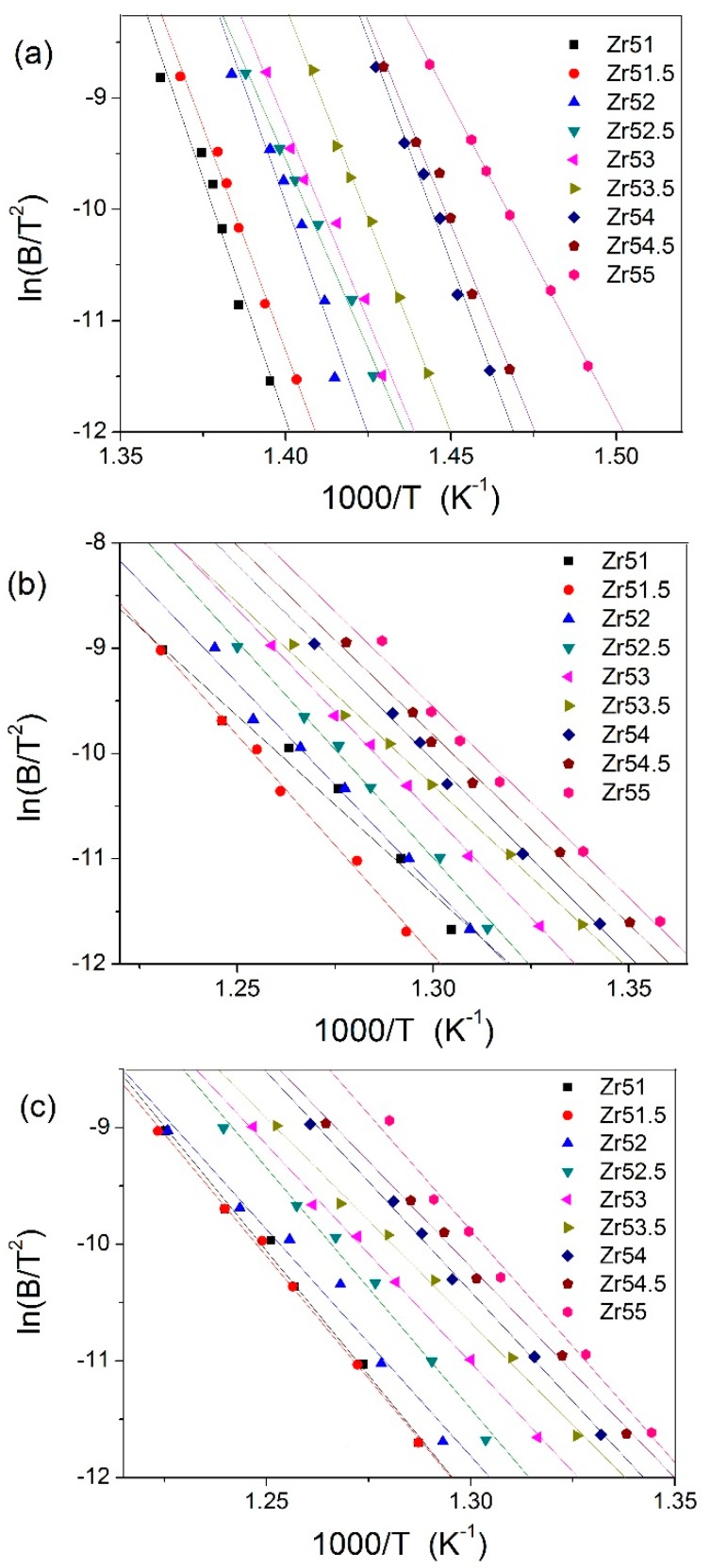
Kissinger plots for: *T*_g_ (**a**); *T*_x_ (**b**); and *T*_p_ (**c**).

The activation energies of *T*_g_, *T*_x_, and *T*_p_ (*E*_g_, *E*_x_, and *E*_p_) are calculated by Kissinger equation [26] and the corresponding fitting curves are presented in Figure 6. It is obvious from Figure 6 that the experimental data can be better fitted by Kissinger equation. The estimated *E*_g_ and *E*_x_ are listed in Table 1.

**Figure 7:**
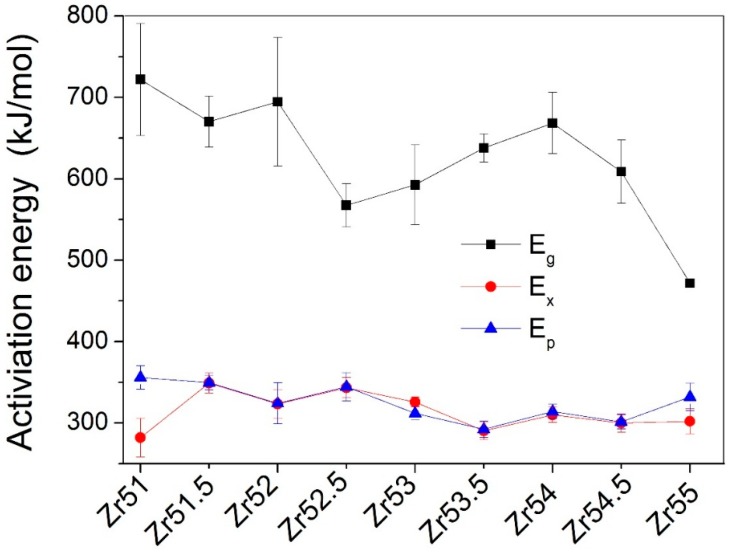
Dependences of the activation energies (*E*_g_, *E*_x_, and *E*_p_) on the Zr content.

The dependences of the *E*_g_, *E*_x_, and *E*_p_ on the Zr content are shown in Figure 7. It is clearly observed in Figure 7 that the dependences of the *E*_g_, *E*_x_, and *E*_p_ on the Zr content are all complex. The *E*_g_ is largest for Zr_51_Al_14.2_Ni_15.9_Cu_18.9_ BMG, while smallest for Zr_55_Al_8.9_Ni_7.3_Cu_28.8_ BMG. The *E*_x_ is smallest for Zr_51_Al_14.2_Ni_15.9_Cu_18.9_ BMG, while largest for Zr_51.5_Al_13.6_Ni_14.9_Cu_20_ BMG. The *E*_p_ is largest for Zr_51_Al_14.2_Ni_15.9_Cu_18.9_ BMG, while smallest for Zr_53.5_Al_10.9_Ni_10.6_Cu_25_ BMG.

**Figure 8:**
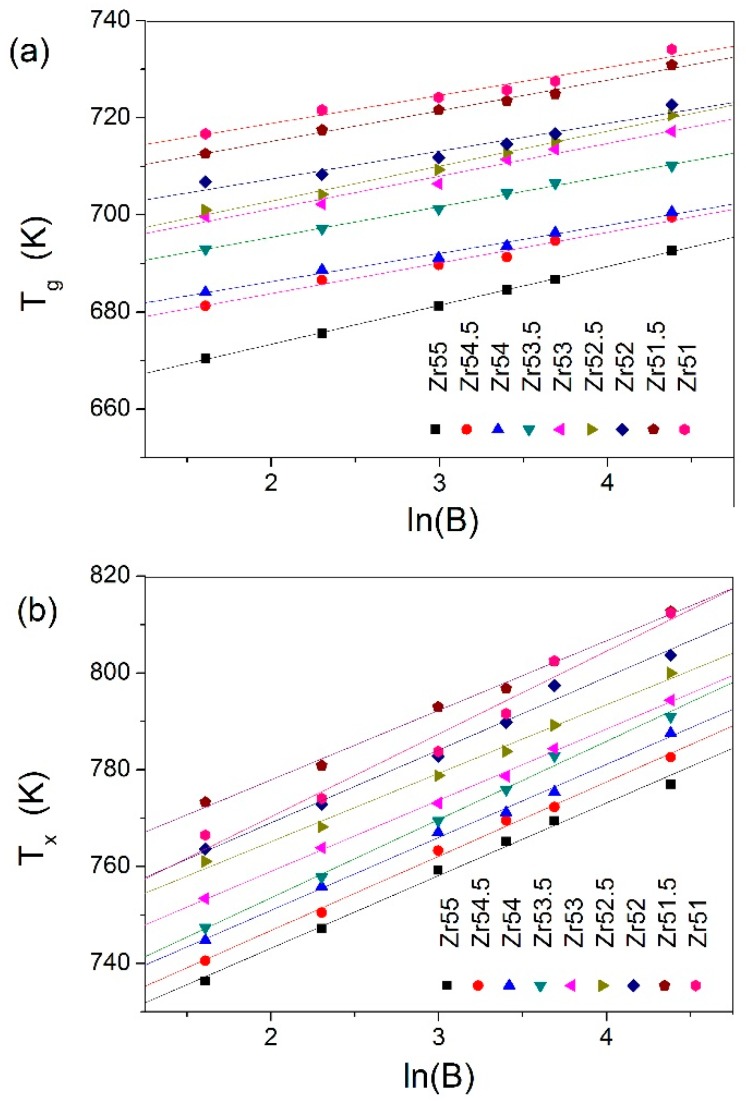

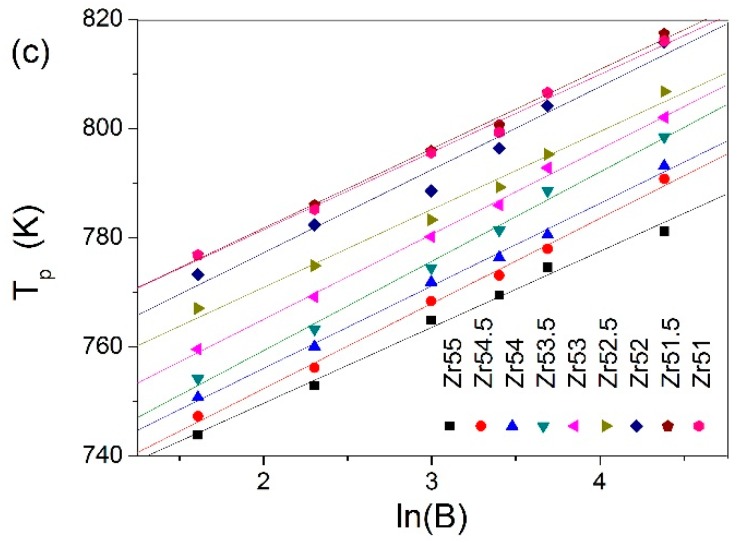
Lasocka plots for: *T*_g_ (**a**); *T*_x_ (**b**); and *T*_p_ (**c**).

In addition, the dependences of the characteristic temperatures (*T*_g_, *T*_x_ and *T*_p_) on the ln*B* (*B* is heating rate) were investigated, as shown in Figure 8. It was found from Figure 8 that the characteristic temperatures (*T*_g_, *T*_x_ and *T*_p_) and the ln*B* can be well fitted by the Lasocka’s equation [27]: T=α+βlnB (*T* is characteristic temperature, α and β are constants). Obviously, β can reflect the sensitivity of the characteristic temperature to the *B*. The higher the β, the more is the sensitivity of the characteristic temperature to the *B*. The β_g_ and β_x_ values are listed in Table 1.

**Figure 9:**
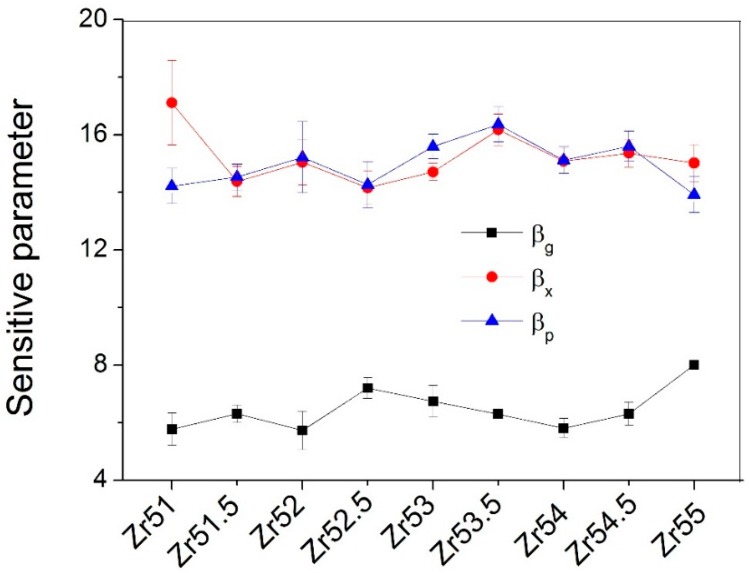
Relationship between the sensitive parameter β and the Zr content.

The dependences of the β values on the Zr content are presented in Figure 9. Both β_x_ and β_p_ are larger than the β_g_, indicating that the crystallization procedure is more sensitive to the heating rate than the glass transition procedure. The β_g_ is smallest for Zr_52_Al_12.9_Ni_13.8_Cu_21.3_ BMG, while largest for Zr_55_Al_8.9_Ni_7.3_Cu_28.8_ BMG. It indicates that the glass transition to the heating rate for the latter has largest sensitivity, while smallest for the former. The β_x_ is largest for Zr_51_Al_14.2_Ni_15.9_Cu_18.9_ BMG, while smallest for Zr_52.5_Al_12.2_Ni_12.6_Cu_22.7_ BMG. The β_p_ is smallest for Zr_55_Al_8.9_Ni_7.3_Cu_28.8_ BMG, while largest for Zr_53.5_Al_10.9_Ni_10.6_Cu_25_ BMG.

**Figure 10:**
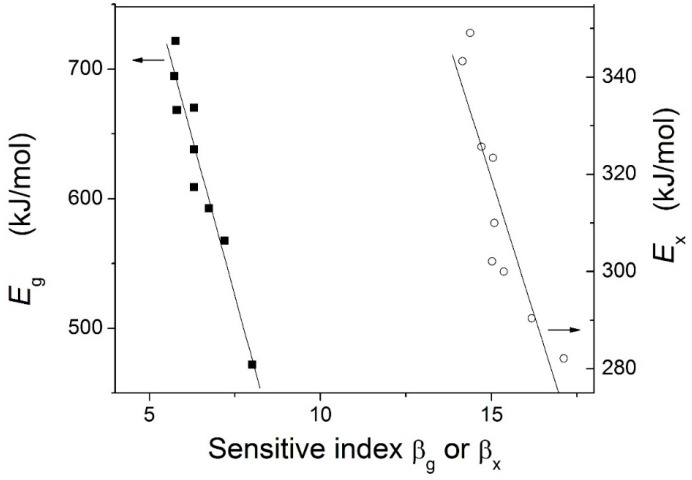
Relationships between the sensitive index and the activation energy.

The relationships between the activation energy and the β were also investigated, as shown in Figure 10. Obviously, the activation energy decreases with increasing β for all studied Zr-based BMGs, which is similar to the results for Zr-Al-Ni-Cu BMGs [28] and Cu-based metallic glasses [29]. Moreover, the liquid behaviors of the alloys were studied by the fragility index *m*. The *m* values of the alloys were estimated by $m=\frac{DT_{g}}{{\left(T_{g}-T_{0}\right)}^{2}\ln10}$ [30] (*D* is the strength parameter, *T*_0_ is the VF temperature, and *T*_g_ is glass transition temperature at a heating rate of 5 K∙min^−1^). The *D* and *T*_0_ are obtained through the best fitting of the tested *T*_g_ values at different heating rates according to the Vogel-Fulcher (VF) relation [30]: $B(T_{g})=A\exp[DT_{0}/(T_{0}-T_{g})]$ (*A* is a constant). The estimated *m* values for these Zr-based BMGs are listed in Table 1. Obviously, the *m* value is largest for Zr_51_Al_14.2_Ni_15.9_Cu_18.9_ BMG, while smallest for Zr_55_Al_8.9_Ni_7.3_Cu_28.8_ BMG.

**Figure 11:**
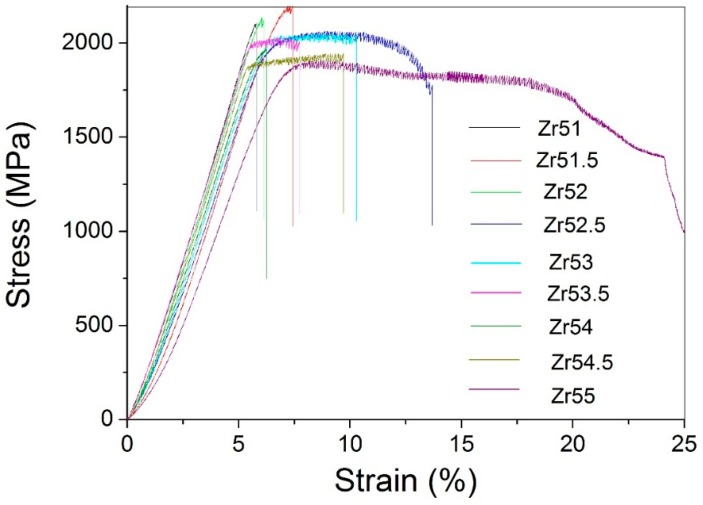
Room-temperature uniaxial compression stress–strain curves for the studied Zr-based BMGs.

Mechanical properties of the studied Zr-based alloys were investigated by room-temperature uniaxial compression tests at a primary strain rate of 1 × 10^−5^ s^−1^. Stress–strain curves are shown in Figure 11 and the corresponding mechanical properties are listed in Table 1. It is obvious from Figure 11 that the plasticity can be clearly observed for all Zr-based BMGs and depends on the composition of the BMG. As shown in Table 1, the plastic strain (ε_p_) is in the region of 0.2%~19.1% for these Zr-based BMGs. The ε_p_ is largest for Zr_55_Al_8.9_Ni_7.3_Cu_28.8_ BMG, while smallest for Zr_51_Al_14.2_Ni_15.9_Cu_18.9_ BMG. The yield strength (σ_y_) can reach up to 2000 MPa for Zr_51_Al_14.2_Ni_15.9_Cu_18.9_ and Zr_52_Al_12.9_Ni_13.8_Cu_21.3_ BMGs whose ε_p_ is less than 1.0%. The fracture strength (σ_f_) is more than 2000 MPa for the BMGs with 51–53 at % Zr. Interestingly, both σ_y_ and σ_f_ are smallest for Zr_55_Al_8.9_Ni_7.3_Cu_28.8_ BMG whose ε_p_ is largest among the studied Zr-based BMGs.

**Figure 12:**
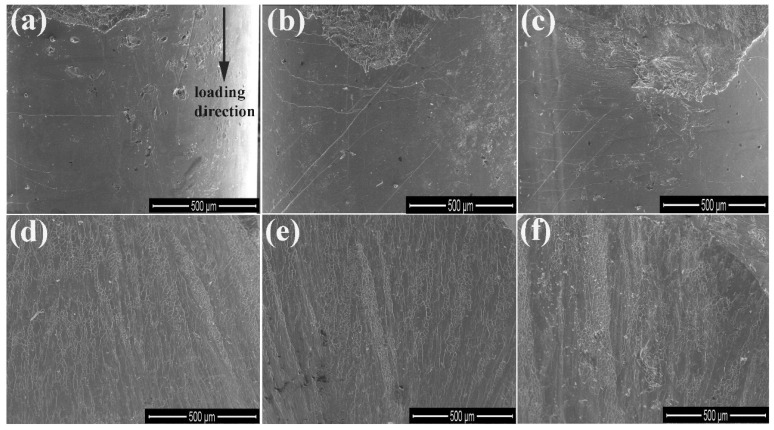
Typical SEM images of side surfaces (**a**–**c**) and fracture surfaces (**d**–**f**): (**a**,**d**) Zr_51_Al_14.2_Ni_15.9_Cu_18.9_, Zr_51.5_Al_13.6_Ni_14.9_Cu_20_, Zr_52_Al_12.9_Ni_13.8_Cu_21.3_, and Zr_54_Al_10.2_Ni_9.4_Cu_26.4_ BMGs; (**b**,**e**) Zr_53_Al_11.6_Ni_11.7_Cu_23.7_ and Zr_54.5_Al_9.6_Ni_8.4_Cu_27.5_ BMGs; and (**c**,**f**) Zr_52.5_Al_12.2_Ni_12.6_Cu_22.7_ and Zr_55_Al_8.9_Ni_7.3_Cu_28.8_ BMGs.

Moreover, fracture surfaces and side surfaces for the fractured BMGs were investigated by SEM. Typical side surfaces for these fractured Zr-based BMGs are shown in Figure 12a–c. The shear bands can be observed on the side surfaces for all Zr-based BMGs. The magnitude and the density of the shear band increase with increasing plasticity. As for the Zr-based BMGs whose ε_p_ is less than 2.0%, the shear bands are scarcely and propagate vertical to the loading direction (see Figure 12a). The intersected shear bands can be observed for the Zr-based BMGs whose ε_p_ is more than 4.0%, as shown in Figure 12b,c. In addition, Figure 12d–f presents typical fracture surfaces for these Zr-based BMGs. The fracture surfaces include smooth regions and vein-like regions for all BMGs. Although the grooves can be clearly observed for all BMGs, the depth and the width of the groove increase with increasing plasticity. More interestingly, the grooves are nearly parallel with each other for the BMGs whose plastic strain is less than 2.0%, while interdigitate each other for the BMGs whose plastic strain exceeds 4.0%.

## 4. Discussion

**Figure 13:**
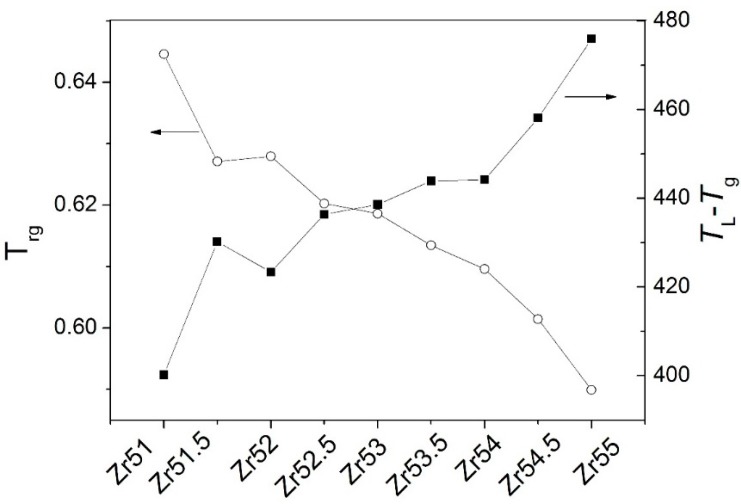
Dependences of the *T*_rg_ and *T*_L_-*T*_g_ on the Zr content.

As shown in Figure 11 and Table 1, there is large different plasticity for the studied Zr-based BMGs. The reasons would be as follows. Firstly, high GFA is advantageous of the homogeneous distribution of the atoms in alloys [17], indicating that the structural heterogeneity in the atomic scale would be difficult for the formation in the BMG with high GFA. The growing results have claimed that the heterogeneity would generally result in good plasticity [3,17,31,32]. In addition, Lu and Liu [33] found that the reduced glass transition temperature *T*_rg_ (=*T*_g_/*T*_L_) is a good indicator for the GFA of the glass forming alloy. The larger the *T*_rg_, the higher is the GFA of the BMG. It is obvious from Figure 13 and Table 1 that the *T*_rg_ nearly decreases with increasing Zr content. It indicates that the GFA decreases with increasing Zr content.

**Figure 14:**
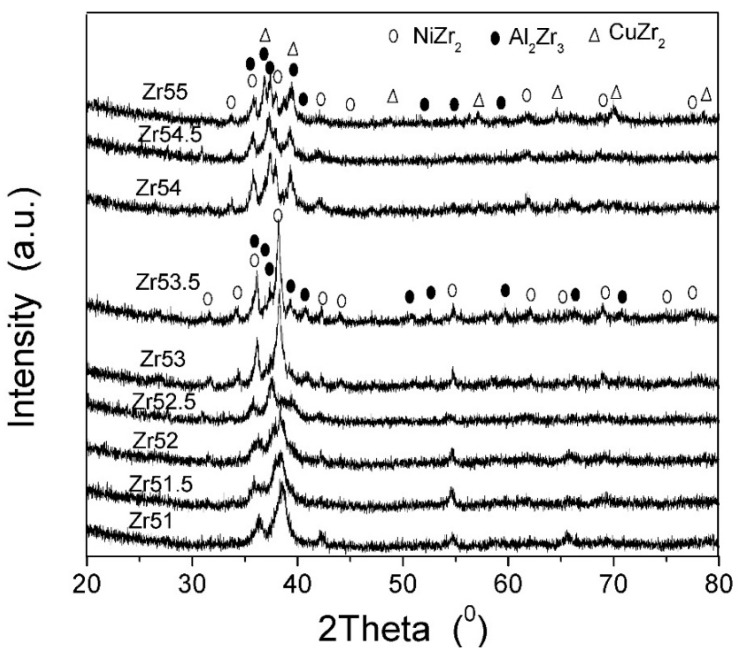
XRD patterns for the studied Zr-based BMGs subjected to DSC test at a heating rate of 80 K∙min^−1^.

It is well known that the GFA of the glass forming alloy originates from the microstructural configuration of the atomic arrangement related with the atomic size, type and interaction among the atoms. The atom radii for Zr, Al, Ni and Cu are 0.162, 0.143, 0.125 and 0.128 nm, respectively. The mixing enthalpy is −23 kJ/mol for Cu-Zr, −49 kJ/mol for Ni-Zr, −44 kJ/mol for Zr-Al, −22 kJ/mol for Al-Ni, −1 kJ/mol for Al-Cu, and +4 kJ/mol for Cu-Ni, respectively [33]. The packing of the atoms in alloys would be changed by the different atom content. Since there is large difference in the mixing enthalpy among atom pairs for the studied Zr-based alloys, the magnitude of short range orders (SRO) would be also changed by varying the atom content in alloys. In addition, there is large negative mixing enthalpy for Zr-Ni, Zr-Al, Zr-Cu and Al-Ni, the SROs corresponding to these atom pairs would be easily formed. XRD analysis (see Figure 14) was performed on the studied BMGs subjected to DSC test at a heating rate of 80 K∙min^−1^ in order to roughly estimate the category and magnitude of the SRO in the studied Zr-based BMGs. A single NiZr_2_ phase can be clearly observed in Zr_51_Al_14.2_Ni_15.9_Cu_18.9_ alloy. A new Al_2_Zr_3_ phase precipitates from the BMG matrix and its magnitude increases with increasing Zr content when the Zr content is less than 52.5%. The magnitude of Al_2_Zr_3_ phase decreases when the Zr content exceeds 52.5%, then increases with increasing Zr content. It would be noted that another new CuZr_2_ phase precipitates from the BMG matrix when the Zr content reaches up to 54%. At the same time, the magnitude of CuZr_2_ phase increases with increasing Zr content. Thus the structural heterogeneity corresponding to the SRO is difficultly formed in Zr_51_Al_14.2_Ni_15.9_Cu_18.9_ BMG, while inversely in Zr_55_Al_8.9_Ni_7.3_Cu_28.8_ BMG. It indicates that the plasticity would be smaller for the former than for the latter (see Table 1).

Secondly, the dwell time in the liquid state can be characterized by $\Delta T=T_{L}-T_{g}$: the larger the ΔT, the longer the dwell time. It would result in the long time for the atomic diffusion, eventually leading to the increase of the probability for the formation of the structural heterogeneity. As shown in Figure 13 and Table 1, the ΔT is largest for Zr_55_Al_8.9_Ni_7.3_Cu_28.8_ BMG and smallest for Zr_51_Al_14.2_Ni_15.9_Cu_18.9_ BMG. Thus, the structural heterogeneity is difficultly formed in Zr_51_Al_14.2_Ni_15.9_Cu_18.9_ BMG, while inversely in Zr_55_Al_8.9_Ni_7.3_Cu_28.8_ BMG. It would lead to larger plasticity for the latter than for the former.

**Figure 15:**
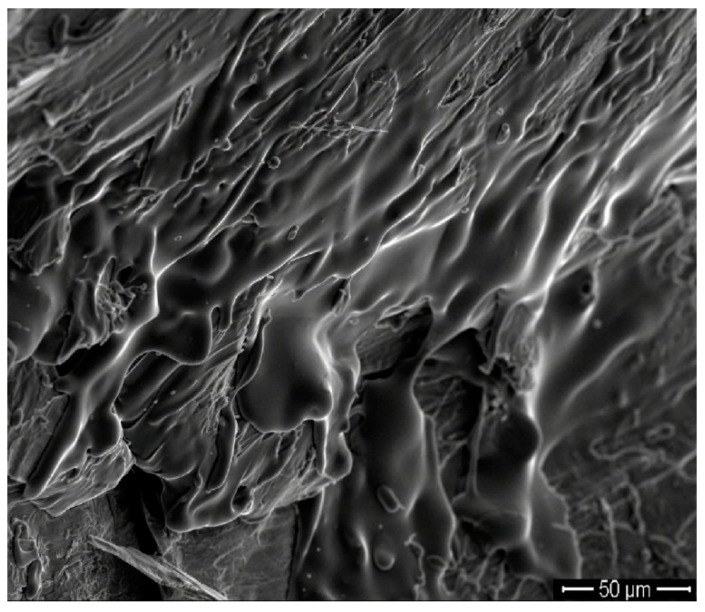
Local SEM image on the fracture surface of Zr_55_Al_8.9_Ni_7.3_Cu_28.8_ BMG.

Thirdly, it is well known that the plastic deformation of the BMG can be regarded as a localized transition from glass to supercooled liquid induced by external stress or temperature increase [17,34]. The external stress can lead to the temperature increase in the shear band [17,35,36,37,38]. The BMGs with low *T*_g_ could be easily gotten into the supercooled liquid region by the external stress and/or temperature. As shown in Figure 15, local melting phenomenon can be clearly observed for Zr_55_Al_8.9_Ni_7.3_Cu_28.8_ BMG and not for the other studied Zr-based BMGs, which is advantageous of the enhancement of the plasticity [36].

**Figure 16:**
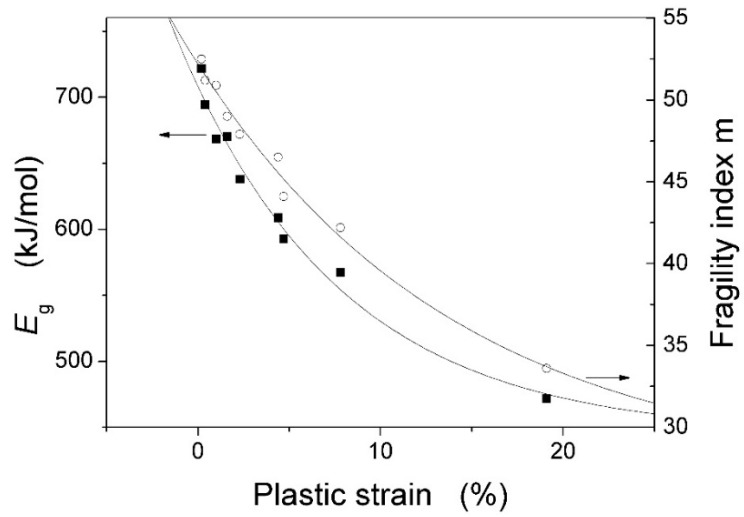
Dependences of the plastic strain on the *E*_g_ and the *m* for the studied Zr-based BMGs.

In addition, the activation energy for the glass transition *E*_g_ is equal to that for plastic flow *E*_p_ [17]. Low *T*_g_ or *E*_g_ would easily lead to yielding or plastic flow of the BMG. As shown in Table 1, both *T*_g_ and *E*_g_ are largest for Zr_51_Al_14.2_Ni_15.9_Cu_18.9_ BMG and smallest for Zr_55_Al_8.9_Ni_7.3_Cu_28.8_ BMG. Interestingly, the ε_p_ increases with decreasing *E*_g_ for the studied Zr-based BMGs, as shown in Figure 16. At the same time, large Δ*T*_x_ indicates good stability of supercooled liquid, resulting in large plastic flow for the BMG. It is clearly seen from Table 1 that the Δ*T*_x_ is largest for Zr_55_Al_8.9_Ni_7.3_Cu_28.8_ BMG and smallest for Zr_51_Al_14.2_Ni_15.9_Cu_18.9_ BMG. Moreover, the *m* value can be considered as the activation energy of the flow for the supercooled liquid and the steepness of the change of the viscosity (η) with the temperature (*T*) at *T*_g_ according to $m={\frac{\partial \mathrm{lg}\eta }{\partial (T_{g}/T)}|}_{T=T_{g}}$ [17]: The smaller is the *m*, the lower is the activation energy and the longer is the time for the flow of the supercooled liquid. That is to say, the small *m* is advantageous of the plastic flow of the metallic glass and would result in large plasticity. As shown in Table 1, the *m* is largest for Zr_51_Al_14.2_Ni_15.9_Cu_18.9_ BMG and smallest for Zr_55_Al_8.9_Ni_7.3_Cu_28.8_ BMG. In fact, it can be clearly seen from Figure 16 that the ε_p_ increases with decreasing *m* for the studied Zr-based BMGs. In a word, the plasticity is largest for Zr_55_Al_8.9_Ni_7.3_Cu_28.8_ BMG and smallest for Zr_51_Al_14.2_Ni_15.9_Cu_18.9_ BMG.

## 5. Conclusions

The studied Zr-Al-Ni-Cu BMGs can be cast into at least Φ8 mm and has a single crystallization event for all heating rates in the studied temperature region. The *T*_g_ decreases with increasing Zr content for all heating rates. Both *T*_x_ and *T*_p_ firstly increase with increasing Zr content and then decrease when the Zr content exceeds 51.5 at % for all heating rates. The Δ*H*_x_ is smallest for Zr_55_Al_8.9_Ni_7.3_Cu_28.8_ BMG, while largest for Zr_52.5_Al_12.2_Ni_12.6_Cu_22.7_ BMG among the studied Zr-based BMGs.

There are two melting procedures when the Zr content is less than 52 at %, while three melting procedures for the other Zr-based BMGs. The distance of two melting procedures is larger for Zr_51_Al_14.2_Ni_15.9_Cu_18.9_ and Zr_51.5_Al_13.6_Ni_14.9_Cu_20_ BMGs than for Zr_52_Al_12.9_Ni_13.8_Cu_21.3_ BMG. The second melting procedure is split into two melting procedures for Zr_52.5_Al_12.2_Ni_12.6_Cu_22.7_ and Zr_53_Al_11.6_Ni_11.7_Cu_23.7_ BMGs, while the first melting procedure is split into two melting procedures for the other BMGs. The *T*_m_ decreases with increasing Zr content when the Zr content is less than or exceeds 52 at %, while inversely for the *T*_L_-*T*_m_. The *T*_L_ is largest for Zr_55_Al_8.9_Ni_7.3_Cu_28.8_ BMG and smallest for Zr_51_Al_14.2_Ni_15.9_Cu_18.9_ BMG among the studied Zr-based BMGs. The *T*_rg_ decreases with increasing Zr content when the Zr content is less than or exceeds 52 at %, while inversely for the *T*_L_-*T*_g_.

The *E*_g_ and *m* are largest for Zr_51_Al_14.2_Ni_15.9_Cu_18.9_ BMG, while smallest for Zr_55_Al_8.9_Ni_7.3_Cu_28.8_ BMG. The *E*_x_ is smallest for Zr_51_Al_14.2_Ni_15.9_Cu_18.9_ BMG, while largest for Zr_51.5_Al_13.6_Ni_14.9_Cu_20_ BMG. The *E*_p_ is largest for Zr_51_Al_14.2_Ni_15.9_Cu_18.9_ BMG, while smallest for Zr_53.5_Al_10.9_Ni_10.6_Cu_25_ BMG. The β_g_ is smallest for Zr_52_Al_12.9_Ni_13.8_Cu_21.3_ BMG and largest for Zr_55_Al_8.9_Ni_7.3_Cu_28.8_ BMG. The β_x_ is largest for Zr_51_Al_14.2_Ni_15.9_Cu_18.9_ BMG, while smallest for Zr_52.5_Al_12.2_Ni_12.6_Cu_22.7_ BMG. The β_p_ is smallest for Zr_55_Al_8.9_Ni_7.3_Cu_28.8_ BMG, while largest for Zr_53.5_Al_10.9_Ni_10.6_Cu_25_ BMG. The activation energy decreases with increasing β for all studied Zr-based BMGs.

The ε_p_ is in the region of 0.2%–19.1% for these Zr-based BMGs. The ε_p_ is largest for Zr_55_Al_8.9_Ni_7.3_Cu_28.8_ BMG, while smallest for Zr_51_Al_14.2_Ni_15.9_Cu_18.9_ BMG. The σ_y_ can reach up to 2000 MPa for Zr_51_Al_14.2_Ni_15.9_Cu_18.9_ and Zr_52_Al_12.9_Ni_13.8_Cu_21.3_ BMGs whose ε_p_ is less than 1.0%. The σ_f_ is more than 2000 MPa for the BMGs with 51~53 at % Zr. Both σ_y_ and σ_f_ are smallest for Zr_55_Al_8.9_Ni_7.3_Cu_28.8_ BMG whose ε_p_ is largest among the studied Zr-based BMGs. The depth and the width of the groove increase with increasing plasticity. The grooves are nearly parallel with each other for the BMGs whose ε_p_ is less than 2.0%, while interdigitate each other for the BMGs whose ε_p_ exceeds 4.0%.

## Figures and Tables

**Table 1:** Glass transition temperature *T*_g_, crystallization temperature *T*_x_, supercooled liquid region Δ*T*_x_, solidus temperature *T*_m_, liquidus temperature *T*_L_, reduced glass transition temperature *T*_rg_ = *T*_g_/*T*_L_, and Δ*T* = *T*_L_ − *T*_g_ at a heating rate of 30 K∙min^−1^, crystallization enthalpy Δ*H*_x_, activation energy for *T*_g_ (*E*_g_), activation energy for *T*_x_ (*E*_x_), sensitivity of *T*_g_ and *T*_x_ to heating rate (β_g_ and β_x_), fragility index *m*, yield strength σ_y_, fracture strength σ_f_, and plastic strain ε_p_.

Metallic Glasses	*T*_g_ (K)	*T*_x_ (K)	Δ*T*_x_ (K)	*T*_m_ (K)	*T*_L_ (K)	Δ*H*_x_ (J/g)	*E*_g_ (kJ/mol)	*E*_x_ (kJ/mol)	*T*_rg_	Δ*T* (K)	*m*	β*_g_*	β*_x_*	σ_y_ (MPa)	σ_f_ (MPa)	ε_p_ (%)
Zr_51_Al_14.2_Ni_15.9_Cu_18.9_	725.7	791.6	65.9	1021.3	1125.9	54.6	721.9	282.1	0.6446	400.2	52.5	5.7692	17.1162	2072.5	2104.9	0.2
Zr_51.5_Al_13.6_Ni_14.9_Cu_20_	723.5	796.8	73.3	1015.0	1153.7	48.4	670.2	349.1	0.6271	430.2	49.0	6.3086	14.3785	1838.0	2184.6	1.6
Zr_52_Al_12.9_Ni_13.8_Cu_21.3_	714.6	789.8	75.2	1060.6	1138.0	51.2	694.6	323.4	0.6279	423.4	51.2	5.7305	15.0479	2041.9	2114.5	0.4
Zr_52.5_Al_12.2_Ni_12.6_Cu_22.7_	712.8	783.8	71.0	1048.7	1149.2	56.7	567.4	343.3	0.6203	436.4	42.2	7.2022	14.1554	1870.1	2040.1	7.8
Zr_53_Al_11.6_Ni_11.7_Cu_23.7_	711.4	778.7	67.3	1044.7	1150.0	50.8	592.7	325.7	0.6168	438.6	44.1	6.7406	14.7100	1840.6	2015.5	4.7
Zr_53.5_Al_10.9_Ni_10.6_Cu_25_	704.5	775.9	71.4	1041.2	1148.4	52.9	637.9	290.4	0.6135	443.9	47.9	6.2987	16.1749	1974.0	1995.4	2.3
Zr_54_Al_10.2_Ni_9.4_Cu_26.4_	693.6	771.2	77.6	1028.2	1137.8	49.6	668.4	310.0	0.6096	444.2	50.9	5.8047	15.0870	1781.6	1959.3	1.0
Zr_54.5_Al_9.6_Ni_8.4_Cu_27.5_	691.3	769.5	78.2	996.4	1149.4	55.4	608.8	300.0	0.6014	458.1	46.5	6.3063	15.3629	1863.1	1907.3	4.4
Zr_55_Al_8.9_Ni_7.3_Cu_28.8_	684.6	765.2	80.6	992.0	1160.5	44.8	471.9	302.1	0.5899	475.9	33.6	8.0065	15.0184	1737.5	1892.6	19.1

## References

[1] Wang W.H., Dong C., Shek C.H. (2004). Bulk metallic glasses. Mater. Sci. Eng. R.

[2] Inoue A., Zhang T. (1996). Fabrication of bulk glassy Zr_55_Al_10_Ni_5_Cu_30_ alloy of 30 mm in diameter by a suction casting method. Mater. Trans..

[3] Liu Y.H., Wang G., Wang R.J., Zhao D.Q., Pan M.X., Wang W.H. (2007). Super plastic bulk metallic glasses at room temperature. Science.

[4] Li Y.H., Zhang W., Dong C., Qiang J.B., Yubuta K., Makino A., Inoue A. (2010). Unusual compressive plasticity of a centimeter-diameter Zr-based bulk metallic glass with high Zr content. J. Alloys Compd..

[5] Hui X., Liu S.N., Pang S.J., Zhuo L.C., Zhang T., Chen G.L., Liu Z.K. (2010). High-zirconium-based bulk metallic glasses with large plasticity. Scr. Mater..

[6] Yokoyama Y., Fujitab K., Yavari A.R., Inoue A. (2009). Malleable hypoeutectic Zr-Ni-Cu-Al bulk glassy alloys with tensile plastic enlongation at room temperature. Philos. Mag. Lett..

[7] Yang Y.W., Hua N.B., Li R., Pang S.J., Zhang T. (2013). High-Zirconium bulk metallic glasses with high strength and large ductility. Sci. China G.

[8] Zhang Q.S., Zhang W., Xie G.Q., Louzguine-Luzgin D.V., Inoue A. (2010). Stable flowing of localized shear bands in soft bulk metallic glasses. Acta Mater..

[9] Hua N.B., Huang L., He W., Pang S.J., Zhang T. (2013). A Ni-free high-zirconium-based bulk metallic glass with enhanced plasticity and biocompatibility. J. Non-Cryst. Solids.

[10] Gan S.F., Chenuo W., Liu Z., Ch K.C., Zhang H.J., Wang J.F., Yu P. (2012). A plastic Ni-free Zr-based bulk metallic glass with high specific strength and good corrosion properties in simulated body fluid. Mater. Lett..

[11] Li H.F., Zheng Y., Xu F., Jiang J.Z. (2012). *In vitro* investigation of novel Ni free Zr-based bulk metallic glass as potential biomaterials. Mater. Lett..

[12] Liu Z.Q., Huang L., Wu W., Luo X.K., Shi M.J., Liaw P.K., He W., Zhang T. (2013). Novel low Cu content and Ni-free Zr-based bulk metallic glasses for biomedical applications. J. Non-Cryst. Solids.

[13] Caron A., Wunderlich R., Louzguine-Luzgin D.V., Xie G., Inoue A., Fecht H.-J. (2010). Influence of minor aluminum concentration changes in zirconium-based bulk metallic glasses on the elastic, anelastic and plastic properties. Acta Mater..

[14] Lee M.H., Lee K.S., Das J., Thomas J., Kühn U., Eckert J. (2010). Improved plasticity of bulk metallic glasses upon cold rolling. Scr. Mater..

[15] Scudino S., Jerliu B., Surreddi K.B., Kühn U., Eckert J. (2011). Effect of cold rolling on compressive and tensile mechanical properties of Zr_52.5_Ti_5_Cu_18_Ni_14.5_Al_10_ bulk metallic glass. J. Alloys Compd..

[16] Tariq N.H., Naeem M., Akhter J.I., Hasan B.A. (2011). Plasticity enhancement in Zr based bulk metallic glass by sand blasting. Mater. Chem. Phys..

[17] Wang W.H. (2012). The elastic properties, elastic models and elastic perspectives of metallic glasses. Prog. Mater. Sci..

[18] Cai A.H., Xiong X., Liu Y., An W.K., Tan J.Y., Pan Y. (2009). Design of new Zr-Al-Ni-Cu bulk metallic glasses. J. Alloys Compd..

[19] Cai A.H., Chen H., An W.K., Tan J.Y., Zhou Y. (2007). Relationship between melting enthalpy Δ*H*_m_ and critical cooling rate *R*_c_ for bulk metallic glasses. Mater. Sci. Eng. A.

[20] Cai A.H., Sun G.X., Pan Y. (2006). Evaluation of the parameters related to glass-forming ability of bulk metallic glasses. Mater. Des..

[21] Cai A.H., Ding D.W., Xiong X., Liu Y., An W.K., Zhou G.J., Luo Y., Li T.L., Li X.S. (2014). Design of Zr-Al-Ni-Cu bulk metallic glasses with network structures. Mater. Des..

[22] An W.K., Ding D.W., Cai A.H., Zhou G.J., Luo Y., Li J.H., Peng Y.Y. (2015). Mechanism, condition and characteristics for the formation of the network structure in Zr-Al-Ni-Cu bulk metallic glasses. Sci. China G.

[23] Cai A.H., Xiong X., Liu Y., An W.K., Tan J.Y. (2008). Artificial neural network modeling of reduced glass transition temperature of glass forming alloys. Appl. Phys. Lett..

[24] Cai A.H., Xiong X., Liu Y., An W.K., Tan J.Y., Luo Y. (2010). Artificial neural network modeling for undercooled liquid region of glass forming alloys. Comput. Mater. Sci..

[25] Cai A.H., Liu Y., An W.K., Zhou G.J., Luo Y., Li T.L., Li X.S., Tan X.F. (2013). Prediction of critical cooling rate for glass forming alloys by artificial neural network. Mater. Des..

[26] Kissinger H.E. (1956). Variation of peak temperature with heating rate in differential thermal analysis. J. Res. Natl. Bur. Stand Sect. A.

[27] Lasocka T.M. (1976). The effect of scanning rate on glass transition temperature of splat-cooled Te_85_Ge_15_. Mater. Sci. Eng..

[28] Cai A.H., Xiong X., Liu Y., Li J.H., An W.K., Luo Y. (2009). Characteristics of near-eutectic and off-eutectic Zr-Al-Ni-Cu glass forming alloys. Mater. Sci. Eng. A.

[29] Cai A.H., Liu Y., Wu H., Ding D.W., An W.K., Zhou G.J., Luo Y., Peng Y.Y. (2015). Phase formation, glass forming ability, mechanical and thermal properties of Cu_50_Zr_50-*x*_Al*_x_* (0 ≤ *x* ≤ 11.0) glass forming alloys. Sci. China Mater..

[30] Yu P., Bai H.Y., Wang W.H. (2006). Superior glass-forming ability of CuZr alloys from minor additions. J. Mater. Res..

[31] Wang J.G., Zhao D.Q., Pan M.X., Shek C.H., Wang W.H. (2009). Mechanical heterogeneity and mechanism of plasticity in metallic glasses. Appl. Phys. Lett..

[32] Du X.H., Huang J.C., Hsieh K.C., Lai Y.H., Chen H.M., Jang J.S.C., Liaw P.K. (2007). Two-glassy-phase bulk metallic glass with remarkable plasticity. Appl. Phys. Lett..

[33] Lu Z.P., Liu C.T. (2002). A new glass-forming ability criterion for bulk metallic glasses. Acta Mater..

[34] Zhao K., Xia X.X., Bai H.Y., Zhao D.Q., Wang W.H. (2011). Room temperature homogeneous flow in a bulk metallic glass with low glass transition temperature. Appl. Phys. Lett..

[35] Zhao M., Li M. (2011). Local heating in shear banding of bulk metallic glasses. Scr. Mater..

[36] Zhang H.W., Subhash G., Maiti S. (2007). Local heating and viscosity drop during shear band evolution in bulk metallic glasses under quasistatic loading. J. Appl. Phys..

[37] Yang B., Liu C.T., Nieh T.G., Morrison M.L., Liaw P.K., Buchanan R.A. (2006). Localized heating and fracture criterion for bulk metallic glasses. J. Mater. Res..

[38] Lewandowski J.J., Greer A.L. (2006). Temperature rise at shear bands in metallic glasses. Nat. Mater..

